# Supplementary material for: Metabolomic and transcriptomic analyses of Fmo5-/- mice reveal roles for flavin-containing monooxygenase 5 (FMO5) in NRF2-mediated oxidative stress response, unfolded protein response, lipid homeostasis, and carbohydrate and one-carbon metabolism
Source: PLoS One. 2023 Jun 2;18(6):e0286692. doi: 10.1371/journal.pone.0286692 (PMC10237457; doi:10.1371/journal.pone.0286692)
Supplement: S2 Table — (DOCX) [file pone.0286692.s002.docx]

**S2 Table2. Plasma metabolite concentrations of 32-week-old *Fmo5^-/-^* and WT mice**

| **Metabolite** | **WT (n=6)** | **KO (n=4)** | ***p* value** |
| --- | --- | --- | --- |
| Total cholesterol | 3.59 ± 0.29 | 2.75 ± 0.05 | 0.035 |
| Triglycerides | 1.115 ± 0.11 | 0.838 ± 0.07 | 0.060 |
| NEFA | 1.11 ± 0.08 | 1.16 ± 0.04 | 0.581 |
| Glucose | 13.29 ± 1.73 | 8.79 ± 0.72 | 0.0007 |

All concentrations are in mmol/L.
